# Mouse Hair Cycle Expression Dynamics Modeled as Coupled Mesenchymal and Epithelial Oscillators

**DOI:** 10.1371/journal.pcbi.1003914

**Published:** 2014-11-06

**Authors:** Ryan Tasseff, Anjali Bheda-Malge, Teresa DiColandrea, Charles C. Bascom, Robert J. Isfort, Richard Gelinas

**Affiliations:** 1 Institute for Systems Biology, Seattle, Washington, United States of America; 2 Procter and Gamble, Mason, Ohio, United States of America; Oxford, United Kingdom

## Abstract

The hair cycle is a dynamic process where follicles repeatedly move through phases of growth, retraction, and relative quiescence. This process is an example of temporal and spatial biological complexity. Understanding of the hair cycle and its regulation would shed light on many other complex systems relevant to biological and medical research. Currently, a systematic characterization of gene expression and summarization within the context of a mathematical model is not yet available. Given the cyclic nature of the hair cycle, we felt it was important to consider a subset of genes with periodic expression. To this end, we combined several mathematical approaches with high-throughput, whole mouse skin, mRNA expression data to characterize aspects of the dynamics and the possible cell populations corresponding to potentially periodic patterns. In particular two gene clusters, demonstrating properties of out-of-phase synchronized expression, were identified. A mean field, phase coupled oscillator model was shown to quantitatively recapitulate the synchronization observed in the data. Furthermore, we found only one configuration of positive-negative coupling to be dynamically stable, which provided insight on general features of the regulation. Subsequent bifurcation analysis was able to identify and describe alternate states based on perturbation of system parameters. A 2-population mixture model and cell type enrichment was used to associate the two gene clusters to features of background mesenchymal populations and rapidly expanding follicular epithelial cells. Distinct timing and localization of expression was also shown by RNA and protein imaging for representative genes. Taken together, the evidence suggests that synchronization between expanding epithelial and background mesenchymal cells may be maintained, in part, by inhibitory regulation, and potential mediators of this regulation were identified. Furthermore, the model suggests that impairing this negative regulation will drive a bifurcation which may represent transition into a pathological state such as hair miniaturization.

## Author Summary

The hair cycle represents a complex process of particular interest in the study of regulated proliferation, apoptosis and differentiation. While various modeling strategies are presented in the literature, none attempt to link extensive molecular details, provided by high-throughput experiments, with high-level, system properties. Thus, we re-analyzed a previously published mRNA expression time course study and found that we could readily identify a sizeable subset of genes that was expressed in synchrony with the hair cycle itself. The data is summarized in a dynamic, mathematical model of coupled oscillators. We demonstrate that a particular coupling scheme is sufficient to explain the observed synchronization. Further analysis associated specific expression patterns to general yet distinct cell populations, background mesenchymal and rapidly expanding follicular epithelial cells. Experimental imaging results are presented to show the localization of candidate genes from each population. Taken together, the results describe a possible mechanism for regulation between epithelial and mesenchymal populations. We also described an alternate state similar to hair miniaturization, which is predicted by the oscillator model. This study exemplifies the strengths of combining systems-level analysis with high-throughput experimental data to obtain a novel view of a complex system such as the hair cycle.

## Introduction

The miniorgan of the hair follicle represents a complex biological system that undergoes repeated phases of death and regeneration over its lifetime [1]–[3]. Understanding of the hair cycle and its regulation would shed light on many other complex systems relevant to biological and medical research including morphogenesis, stem cell biology, response to environmental perturbations and general spatiotemporal patterning [4]. The stages of the hair cycle have been well documented, at least from a morphological standpoint, in mouse models [5]. The period of hair growth, known as anagen, involves rapid proliferation of follicular epithelial cells, such as MatriX (MX) cells in the hair bulb, which surround a key group of mesenchymal cells that form the dermal papilla (DP). Matrix cells differentiate to eventually compose various epithelial populations of the hair shaft. Anagen is followed by catagen, which is characterized by high levels of apoptosis. Finally, telogen is typically described as a quiescent period between growth phases.

The molecular mechanisms underlying this cyclical pattern of death and renewal in hair follicles are not well understood; however, some general concepts, as well as specific molecular regulators, have been identified. One key aspect is the communication between epithelial and mesenchymal cells. Numerous studies have identified physical interactions between these cell populations, as well as several possible signaling molecules [6]. One well studied signaling molecule of the hair cycle is Tgf*β*2, which is synthesized and secreted by DP cells. The evidence suggests that, in general, Tgf*β*2 suppresses proliferation and induces catagen-like changes in the follicle, including apoptosis of MX cells [7]. However, recent studies have identified a Tgf*β*2 mediated pathway which activates epithelial stem cells to promote hair follicle regeneration [8]. This underscores the complexity of the signaling pathways involved.

Mathematical models of general features of hair cycling have also been studied. In a recent study by Murray *et al.*, the authors model follicle growth and coupling as an excitable medium [9]. Their model incorporates general aspects of hair cycle regulation, and shows qualitative agreement to experimental observations. Also recently, Al-Nuaimi *et al.* developed a general model for hair cycling based on observations in the literature [10]. These authors derived a mathematical, kinetic model which proposed that negative feedback between dynamic MX keratinocytes and static DP cells could reproduce the cyclical growth patterns of the hair follicle. Although these models are significant, they do not attempt to incorporate any specific molecular details in a data-driven approach by formally analyzing large scale experimental data sets. In the study by Lin *et al.*, mRNA microarrays were compiled over the first three rounds of hair growth: morphogenesis, the second naturally synchronized cycle and a depletion-induced cycle [11]. The results demonstrated recurrent gene expression corresponding to hair growth, and the authors specifically focused on genes related to circadian rhythms. However, the study does not address the many other genes observed to have similar patterns. Currently, an unmet need is the development of data-driven approaches that can couple the existing transcriptome-wide data to systems-level properties of hair cycling using formal dynamic models.

Nonlinear dynamical models have provided valuable insights into many oscillating biological systems [12]–[14], and have even been used to suggest general design principles of oscillating metabolic and signaling networks [15]. In general, simplified oscillator models have been developed to describe properties resulting from oscillator interactions or coupling. One such model is the well-studied Kuramoto model [16]. Here all oscillators are interconnected with the same coupling strength, and studied as a single mean field. Although a major simplification, mean field models have successfully described high level properties of many large, complex systems including statistical mechanics (for a review see [17]), economics [18], [19] and even social networks [20]. Importantly, the Kuramoto model is capable of capturing a critical phase transition from an incoherent state to one in which all oscillators converge to a single, coherent cluster. This behavior is referred to as synchronization and is quantified by complex order parameters [21]. Modified and extended versions of the Kuramoto model have been used in many complex systems [22] including synthetic genetic networks [23]; cyclical gene expression and cellular networks [24]; neural networks for memory and brain activity [25], [26]; chemical oscillators [16]; and laser arrays [27], [28]. We would not expect such models to be capable of incorporating or identifying mechanistic molecular interactions or specific details; however, given the above literature evidence, they can be quite successful at describing systems level properties, such as synchronization, and the sufficient, underlying conditions that can produce them.

Our aim here was to investigate a subset of genes whose expression changes as a function of time in a potentially periodic manner, similar to the cyclical nature of hair growth. Previous modeling studies, which have focused on general aspects of hair growth, represent important initial steps in applying mathematical strategies to understanding the hair cycle [9], [10]; however, these models are not driven by molecular-level data. In contrast, other studies use high-throughput molecular-level data to identify important targets, and they apply additional experiments to delineate specific molecular mechanisms [11]; however, these studies are limited to investigation of a small number of genes, and they do not attempt to place the observations into a quantitative modeling context. In this study, we focused on two complementary mathematical modeling strategies that look at high-level features, such as average dynamic behavior, that is based on the individual patterns of thousands of genes. Thus, we are attempting to bridge the gap between the two strategies described above. Using whole skin, transcriptome-wide expression data, we demonstrate the existence of two subsets of genes that have synchronized, out-of-phase expression profiles. Motivated by this observation, we applied a coupled oscillator modeling framework to identify a specific coupling configuration that spontaneously, and stably reproduced the observed synchronization. We then applied a 2-population mixture model to associate the corresponding gene clusters to two computationally determined populations, a rapidly expanding population and a relatively static background population. The estimated population dynamics indicated an association between computationally derived background/expanding populations and the mesenchymal/follicular epithelial cells, respectively. Cell type specific enrichment analysis and experimental imaging with *in situ* hybridization and immunofluorescence all demonstrated similar associations. The results describe a coupling scheme, between these two cell populations, which would be sufficient to maintain the observed synchronization. Specific signaling molecules were also identified as being priority follow-up targets for drivers of synchronization. To our knowledge this is the first attempt at integrating high-throughput molecular data with a mathematical model to predict systems level properties, such as synchronization and population dynamics.

## Results/Discussion

### Identification and characterization of periodic expression signals

Given the proposed cyclical nature of hair growth, we investigated the possibility of periodically expressed mRNA in the microarray data collected by Lin *et al.* [11]. We assumed that such expression patterns may relate to hair cycle regulation. We applied a periodic identification scheme for non-uniformly sampled data [29]. This method estimates a discrete Fourier Series Decomposition (FSD) for each expression signal by robust regression. We identified 4627 probesets (mapping to 3567 unique genes) as significantly periodic signals with a false discovery rate of 10%. Using the semantic measure Normalized Google Distance (NGD), we found that 315 of the corresponding periodic genes had a notable proximity to the hair cycle described in a survey of PubMed abstracts. This translates to an enrichment p-value of 2.5E-5. For example, periodic genes with the lowest NGD are discussed in the literature as being related to hair pigmentation (Mc1r, Tyrp1, Stx17), growth and cycle regulation (Liph, Foxn1), disorders and malformations (Lpar6, Zdhhc13, Krt85) and general associations to hair (Krt28). For a full list of genes and related NGDs to hair see Supplementary File S1.

To further investigate the periodic expression, we assigned a specific frequency and a phase shift to each signal. This was done using the Principal Periodic Component (PPC) as an approximation to the FSD. Both the PPC and FSD reasonably recapitulated the time course trajectories, primarily the low frequency expression signals (Supplementary Figure S1 top). Furthermore, the majority of the periodic signals, 3988 probesets, were associated to this low frequency, which corresponds to a period of 31 days (Supplementary Figure S1 bottom). The 31 day period of expression was on the same time scale as the hair cycle, and further suggested a relationship between the corresponding genes and hair growth and regulation. Although repeating cycles were not directly observed to demonstrate cyclical behavior, we note that the data was a composite of both the second natural and depilation-induced hair growth cycles and, therefore, the expression patterns were common to, at least, these two cycles.

**Figure 1:**
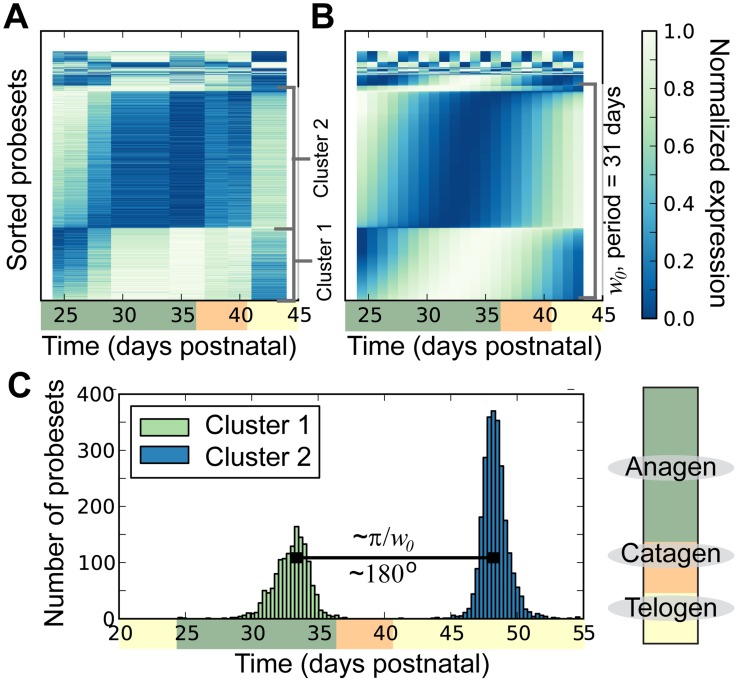
Periodic gene expression in mouse hair cycle. An approximation of the hair cycle phase corresponding to the time scale is indicated via a color bar, see legend in lower right. (**A**) Heat map of actual expression data for probesets identified as periodic. For visualization, the data is normalized relative to the corresponding maximum and minimum values (this normalization was not used in statistical analysis), and sorted first by principal frequency and second by phase. (**B**) Heat map of expression estimated by the principal periodic component corresponding to A. Sorted and normalized as in A. (**C**) Histogram of the phases for probeset expression patterns corresponding to the longest period, 31 days, estimated by the principal periodic component.

For visual examination, we sorted the periodically expressed probesets by frequency and phase shift (Figure 1A). We compared this data to the PPC trajectories (Figure 1B) to underscore the similarities. We noted a distinct clustering of the expression signals, including two clusters within the low frequency probesets. The lower and upper clusters demonstrate maximal and minimal expression near the end of anagen, respectively. This reciprocal periodic behavior is referred to as out-of-phase periodicity. We calculated the phase shift based on the time to the next maximum value. The phase shift (Figure 1C) clearly identifies these two tightly clustered groups. We associated 1452 probesets to cluster one and 2536 to cluster two. Cluster one and two correspond to probesets that are predicted to reach maximum expression at approximately 33 (first anagen phase after morphogenesis) and 48 (following telogen phase) days postnatal, respectively. The separation of the phase shift further indicates that the two groups are almost exactly out-of-phase. The mean of the two groups is separated by 15.4 days which in polar coordinates corresponds to approximately 180°. Although fast cycling genes were identified, we chose to overlook this group due to a relatively poor fit to the PPC, median coefficient of determination was less than 0.5 (Supplementary Figure S1 Top). However, given that the data was derived from full-thickness mouse skin, it is possible that cyclic gene expression in Keratinocytes could be contributing to the short period signal. This possibility was strengthened with the recent report that human epidermal stem cell functions are regulated by circadian oscillations [30] with a period of 24 hours in vitro. Additional experiments, with higher time-resolution sampling, may better describe fast cycling genes and may provide a link between Keratinocytes, hair-cycling and circadian rhythms.

**Figure 2:**
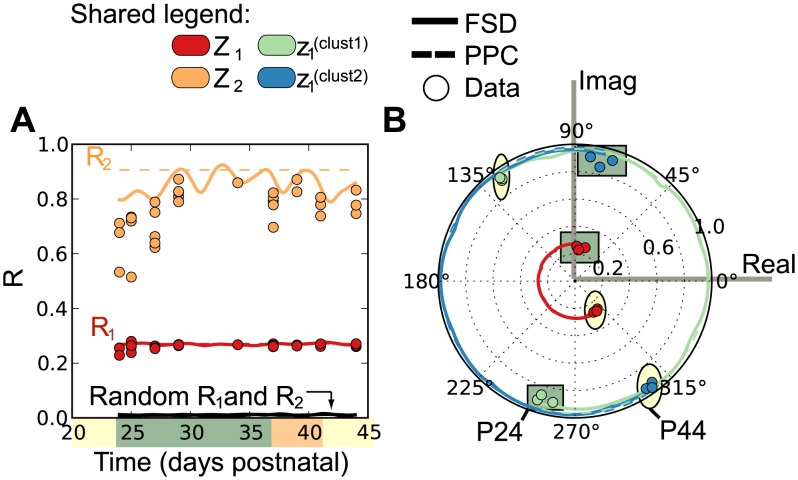
Complex order parameters for the hair system. Here we only consider the low frequency expression patterns. Different levels of approximation were used to calculate the instantaneous phase, Fourier Series Decomposition (FSD), the Principal Periodic Component (PPC) as well as a combination of FSD and data referred to as Data. (**A**) The magnitude of the first (red) and second (orange) order parameter. Note that all approximations for 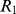 are similar and that both 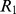 and 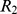 are much higher then random (shown in black). (**B**) The first order parameter for the full system (red, corresponds to A) as well as sub systems cluster one (green, corresponds to Figure 1C) and cluster two (blue, corresponds to Figure 1C). Time points corresponding to anagen postnatal day 24 (P24) and telogen postnatal day 44 (P44) shown in green squares and yellow ovals, respectively. Note that the clusters are highly synchronized, with radii near one, and angles 180° apart.

The clustering of periodic signals was quantified using complex order parameters [21], [31], [32]. Considering only the periodic component of the low frequency expression signals corresponding to a 31 day period, we can visualize the expression as points moving around the unit circle in the complex plane as they travel through the cycles. How tightly grouped these points are can be quantified by a set of order parameters, 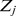:
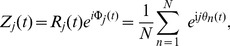
(1)where 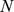 is the number of oscillators and 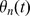 is the instantaneous phase or the position of oscillator 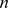 on the unit circle at time 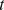. See Methods for more details on the formulation of EQ 1. When studying systems of coupled oscillators, the magnitude of 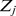, denoted 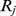, is used to quantify the coherence or synchronization of the system (Supplementary Figure S2 shows typical 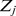 values for specific configurations of points). In such systems, a high level of synchronization is typically the result of coupling between oscillators. The low frequency expression signals in the hair cycle demonstrated high out-of-phase synchronization as measured by 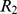, as well as a notable asymmetry, due to uneven sized clusters, measured by 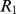 (Figure 2A). As a negative control, we randomized the oscillator phases to demonstrate 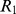 and 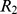 near zero for a similar, but un-clustered system (Figure 2A, black lines). Synchronization was further exemplified by considering order parameters calculated for the specific clusters, denoted 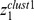 and 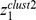 (Figure 2B, green and blue lines corresponding to clusters shown in Figure 1C). This level of synchronization appears to be dynamically stable throughout the time course. If we can expect similar molecular behavior underlying subsequent cycles of hair growth, we would anticipate these periodic expression signals to repeat. In an ideal case the low frequency gene expression could then be viewed much like a system of oscillators, and the observed dynamic stability could be investigated in that context.

### Expression modeled as a system of coupled oscillators

We believed that strong synchronization over multiple expression signals was indicative of regulation between the corresponding genes. As mentioned above, systems of multiple agents with periodic behavior are often described using coupled oscillator models. Furthermore, and most significantly, we found a striking similarity between the observed expression in the mouse hair cycle and a simple system of coupled oscillators formulated by Hong and Strogatz [33]. In this model one possible attractor (or long time behavior) was the synchronization of two, asymmetric, out-of-phase, oscillator clusters. Spontaneous, stable synchronization was observed when one cluster was positively coupled to the system's macroscopic rhythm, embodied by complex order parameter 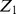 (see EQ 1), and the other was negatively coupled. In this case positive or negative coupling indicates oscillators that move towards or away from 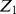, respectively. Although such models are a significant simplification from the true biology, the qualitative agreement was encouraging, and we wished to investigate if reasonable insights could be drawn from such an abstraction. In the following we consider a system of Low Frequency Oscillators (LFO) corresponding to the 31 day period expression signals identified above.

**Figure 3:**
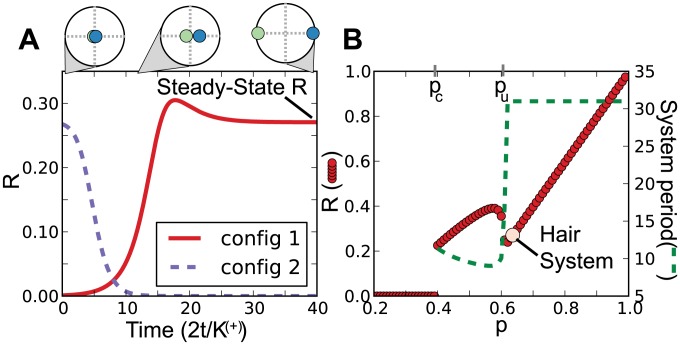
Simulation results from mean field coupled oscillator model. All curves are calculated by solving EQ 2 (for additional details also see EQ 11). The magnitude of the first order parameter, 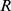 shown in red, can be easily calculated from the individual order parameters, 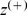 and 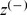. Here, 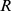 is related to the first order parameter in Figure 2, also shown in red (note the subscript was dropped for convenience). (**A**) Simulation results of 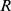 for configuration one (config 1, solid) and configuration two (confg 2 dashed). Here config 1 relates to cluster one having negative coupling (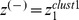). Note that the synchronization was stable only in config 1. We also show the incoherent result when configuration two (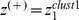) was set near, the steady-state value. The top plots show the values of 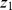 for both cluster 1 (green) and 2 (blue) on the unit circle at time = 1, 12 and 40 days. Note that the clusters are out-of-phase. A movie of the individual oscillators corresponding to configuration 1 is available as Supplementary file S2. (**B**) A simulated bifurcation analysis of the model showing the stable attractors for 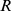 at different values of 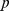 (red dots). We note that the simulation results agree with the analytical results of 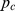, loss of the incoherent state, and 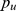, the upper bound of the wave state. The estimated period of the hair cycle is shown by the dashed line. The values corresponding to the observed hair system are highlighted, note that it is near a critical change in 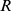 that corresponds to a sharp decrease in the period.

We first considered if our system could be quantitatively described by the above model. At the level of individual oscillators this model can be written as
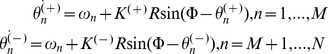
(2)where 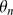 is the phase of the 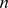
th oscillator, 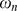 is the natural or intrinsic frequency for 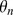, 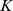 is the coupling constant, 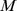 is the number of positively coupled oscillators, 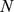 is the total number of oscillators, 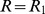 and 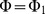 from EQ 1 where the subscript is dropped for simplicity. The dot denotes change with respect to time. Here, we have assumed two groups representing positive and negative coupling denoted by superscripts + and −, respectively. The coupling constants are related by 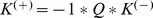 where 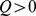, 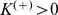, 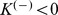. This model can be simplified to two dimensions when describing only the dynamics of the first order parameter for the two clusters (recall Figure 1C, green and blue clusters), which was what we focused on here as a high level characterization of the system
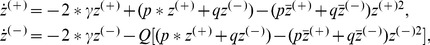
(3)where 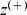 and 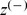 are the first order parameters (similar to EQ 1 with 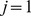) for the two oscillator groups related to positive and negative coupling, respectively; 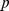 is the proportion of positively coupled oscillators and 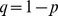. The bar denotes complex conjugate. Given the first order parameters of our observed clusters (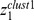 and 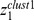, recall Figure 2B green and blue lines) and the number of oscillators for the two clusters (which provides 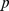), we solved exactly for the relative coupling strength, 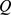, and the intrinsic frequency distribution, 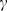. We refer the reader to Methods for a detailed description of this process including additional assumptions. Interestingly, the only stable configuration was negative coupling of cluster 1, the smaller cluster (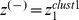). Figure 3A shows that this configuration resulted in spontaneous, stable synchronization (config 1, red solid line, a corresponding movie of the individual oscillators is available in Supplementary File S2), a steady-state magnitude of the first order parameter equivalent to the observed magnitude (recall Figure 2A), and clusters that remain 180° out-of-phase (upper panel of Figure 3A). Furthermore, if cluster 1 is positively coupled (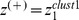), the model system is unstable and no synchronization is observed (Figure 3A purple dashed line, config 2). The results suggest that a positive coupling of cluster 1 to the system is physically unlikely.

This result provides us with our first biological insight, specifically, the genes corresponding to cluster 1 were negatively coupled to the system's macroscopic rhythm, and repelled by this average behavior of the system. If these genes can be associated with specific cell populations, then inhibition or repression of this population by the system would explain the simultaneous negative coupling of a large number of genes. Inhibition at the cellular level could be achieved by regulated apoptosis, which is consistent with a previous model of the hair cycle from the literature [10]. The authors described that negative feedback in the form of regulated apoptosis or inhibition of regulated proliferation could produce observed cyclical hair growth patterns.

Using this coupled oscillator model, we investigated what other states could be possible if specific variables were changed. We constructed a bifurcation diagram that shows the steady-state behavior of the system given different values of 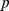 (see EQ 3) and assuming that other properties remain constant. Figure 3B shows three qualitatively different states for varying values of 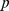. Low values of 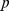 relate to an incoherent state which has no synchronization and, therefore, no clustering. For large values of 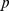, we find the 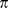-state in which two clusters are stable, out-of-phase and oscillate with the same long period throughout a range of 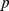 values, with increasing asymmetry to one cluster. Finally, we observed a traveling wave state for intermediate values of 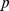, here the period of the system is greatly reduced and even variable with respect to 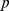. These states were described by Hong and Strogatz [33]. Interestingly, the hair system lies at the edge of the 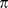-state, near a bifurcation into the traveling wave state. A reduction in 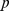 corresponds to a decrease in the relative size of cluster 2, and would reduce the effective negative coupling on oscillators in cluster 1, which is what drives the system into a different state. Therefore the model anticipates that on average, removal of oscillators in cluster 2 would result in a loss of regulation which is specifically associated with varying, high frequency oscillations. Furthermore, we noted that the reduced period of the traveling wave state is similar to the fast cycling of short hairs in an existing model of hair cycle [10], which was shown to result from changing parameters associated with negative feedback. This alternative state may be biologically related to the pathological state of hair miniaturization and androgenic alopecia.

Although our model recapitulated the observed synchronization, we emphasize here the various abstractions introduced. First, we did not attempt to model the physical, molecular interactions involved, as we felt such an approach would be too error prone given limited data and *a priori* knowledge [34]. Instead, we chose to implement the simplest phenomenological model we could conceive to describe observed behaviors. Here oscillators represented probesets with periodic expression patterns (see Methods EQ 7). Modeled oscillators changed due to two terms: the intrinsic term, 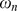, and the coupling terms, 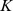 (both from EQ 2). In a physical model the intrinsic term would represent some constant, external force that independently drives oscillations, in this case the intrinsic term represents all the unknown interactions that were the basis for periodic expression. As a result, insight and prediction related to these interactions was beyond this model's reach. In contrast the coupling terms were of most interest, and represented the average force felt by oscillators of a certain group, due to other oscillators. This coupling term is what drove the observed out-of-phase synchronization. The model was further abstracted as we specifically considered the average dynamics of the two clusters (see EQ 3), which were coupled via the average coupling of the underlying oscillators. In a sense the model treated interactions as being ‘smeared-out over all oscillators. Although exact physical interpretation is difficult, this average effect on the many corresponding genes may be the result of an extensive transcriptional program or a cellular event such as regulated apoptosis or proliferation, and suggests that these clusters may be related to specific cell populations.

### Associating gene clusters to biologically relevant cell populations

Given previous results, we hypothesized that the two observed clusters of LFO gene expression may relate to specific cell types or general cell populations. We noted that observed relative expression changes can be attributed to changes in relative cell population size as opposed to intracellular changes. Furthermore, opposite expression patterns can result when one population is constant in size and the other is changing (See Supplementary Figure S3, and Methods for details). To investigate further, we applied *in silico* microdissection [35] to estimate properties of two distinct cell populations from the heterogeneous hair cycle data. Here, the 2-population mixture model assumes static intracellular gene expression. Furthermore, we also assume one population is expanding, while the second is a static background population. We emphasize here that we make no assumption as to the specific cell types contributing to these populations nor the relative expression levels of genes within these populations. We then fit this model using all of 45k+ probesets from each micro array chip.

**Figure 4:**
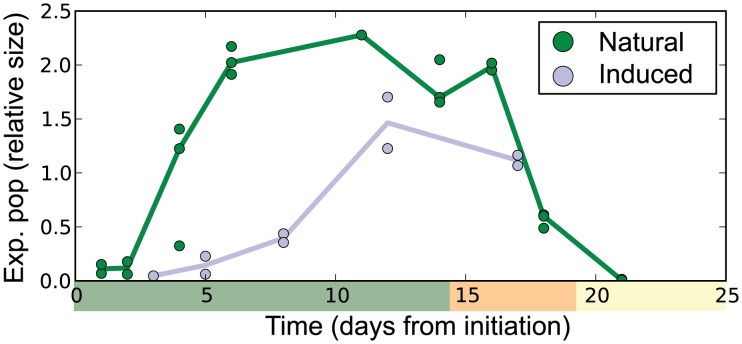
Predictions on population dynamics determined by the two population model. The predicted relative size of the expanding population for both the natural (green) and induced (light gray) hair cycle expression data. The time scale used was set relative to initiation, which was after morphogenesis (postnatal day 23) or after depletion for the natural and induced cycles, respectively.

From the heterogeneous hair cycle samples, we were able to approximate the dynamics of dominant expanding cell populations (Figure 4). The trajectory was consistent with cells associated with rapidly proliferating follicle epithelial cells. In particular the model estimated (without any *a priori* knowledge) a sharp and complete depletion of the expanding population within the catagen time frame. The model also identified differences between samples from the natural and induced cycle. Specifically, the model estimated a slower anagen onset in depletion induced mice, this was also observed by comparing morphologies of tissue sections in Lin *et al.* [11]. Furthermore, we demonstrated that these features were not estimated in a negative control (see Supplementary Figure S4), using a permutation strategy to simulate data with no hair cycle relationship. This suggested that our results were not due to an artifact or bias introduced in the analysis. These observations suggested that the *in silico* microdissection procedure was able to identify expanding cell populations compared to a static background populations.

**Figure 5:**
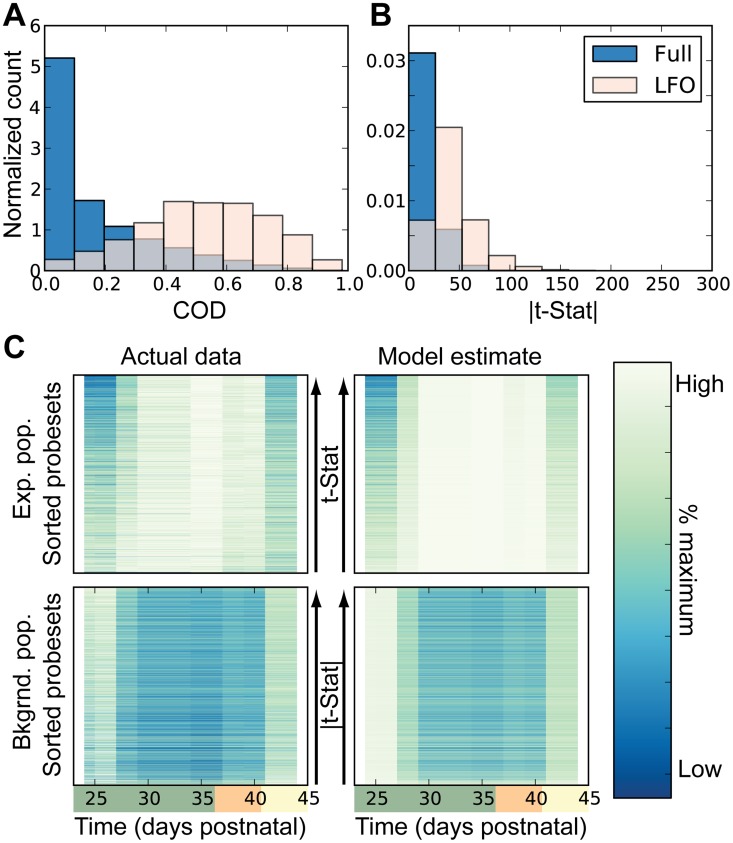
Predictions on expression dynamics determined by the two population model. (**A**) Histogram of the coefficient of determination (COD) for model estimated expression, shown for all probesets (dark blue background) and probesets identified as low frequency oscillators (LOF, light pink foreground). (**B**) The magnitude of the t-statistic used to estimate differential expression between the two estimated populations, shown for all probesets (dark blue background) and probesets identified as low frequency oscillators (LOF, light pink foreground). (**C**) Normalized expression data, 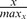, for the two model populations. The left column shows actual expression data and probesets are ordered by the magnitude of the t-statistic. The right column shows expression estimated by the model ordered as in left column.

The model was also able to estimate static intracellular expression levels for each population. Combining this with the estimated population size, we were able to compare estimated expression levels to those observed in the data. Overall, we found that the majority of expression signals were not well described by such a simple model (Figure 5A, blue histogram); however, the model was able to describe the majority of the variation in the expression of the LFOs (Figure 5A, pink histogram, 50% of probesets demonstrated a COD>0.5). As above we did not identify such improved statistics of the LFOs in the permuted negative control. This suggested the significance of such a model in describing expression of the LFO subgroup. Using the estimated expression and standard error estimates, we identified probesets with statistically significant differential expression between the two estimated populations (expanding and background) in the form of a t-statistic. The t-statistic shows the expression difference relative to the estimated error. Again more statistically significant differential expression was found in the LFO subgroup (Figure 5B). Probesets from the LFO subgroup were assigned to the population in which they demonstrated a statistically significant increase in expression, 99.7% of the LFO probesets met the statistical requirements for assignment. Figure 5C shows the remarkable similarity between clusters 1 and 2 (recall Figure 1C) and the expanding and background population respectively. In fact, dividing the LFO probesets in this manner yields the same division as achieved when considering oscillator phase (Supplementary Figure S6). Ultimately, this procedure allowed us to associate probesets and corresponding genes to two dynamically distinct populations.

We next investigated the possibility that the estimated populations were associated to specific biological cell types involved in the hair cycle. We made use of two existing studies, Rendl *et al.* [36] and Greco *et al.* [37], which dissected hair follicles into specific, predefined cell types. For each cell type, signature genes, genes expressed predominantly in that cell type, were identified. We then calculated the enrichment of these signature genes in the two estimated populations (Supplementary Table S1). In particular, we found a significant enrichment for epithelial MX and, to a much lower degree, ORS cells in the expanding population, 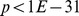 and 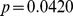 respectively, but not for the background population. Alternatively, we found a significant enrichment for mesenchymal DP cells in the background population, 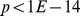, but not for the expanding population. Other cell types, Melanocytes and Bulge Cells, were found to be significantly enriched in both populations, but to a larger degree in the background population (see Supplementary Figure S7A for relative signature list size and overlap with estimated populations). We also observed overlap between the experimentally determined gene signatures, for example the overlap between Bulge [37] and DP [36] signature genes (see Supplementary Figure S7B) corresponds to an enrichment p-value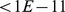. The results show that the computationally estimated populations do not uniquely represent a specific, predefined cell type; however, they do have distinct associations. Furthermore the association of epithelial MX and ORS cells to the rapidly expanding population and DP cells to the static background population was consistent with the known relative population dynamics of those cell types. We emphasize that DP population size does change throughout the hair cycle [38]. However, the DP population is not observed to undergo the enormous expansion and apoptosis characterizing hair epithelial cells [39]–[41]. Thus, conceptually the DP may be considered considerably less dynamic than the MX derived population [2], which is consistent with our findings here. For a full list of significant probesets with the t-statistic indicating population association, and other metrics, see Supplementary File S3.

These results provide us with a second biological insight: the genes in LFO cluster 1 were associated with expanding cell populations of the follicle that were enriched for follicle epithelial cells as defined Rendl *et al.* [36], and cluster 2 was associated with background populations that were enriched for mesenchymal DP cells. This bolsters our previous hypothesis that an inhibitory, possibly apoptotic mechanism, is acting on the expanding, epithelial cell population, and that this mechanism is involved in synchronized gene expression of the hair cycle.

### Experimental follow-up on expression data and associations to distinct cell populations

To verify specific gene expression patterns and to investigate the localization of gene products within the hair follicle, we applied qRT-PCR, *In Situ* Hybridization (ISH), and protein antigen staining. To generate tissue samples, we aligned the hair cycle in 10-week old mice with the shave/depilatory induction protocol. Two animals were sacrificed for each of the five time points considered; however, qRT-PCR was done using four technical replicates for both samples and imaging shows results typical of multiple follicles observed over the two biological replicates. Phenotypic changes were quantified by melanogenesis scoring, which is known to correspond to active hair growth. Both biological replicates were observed to have entered anagen within 16 days of induction and to have completely finished the first round of post induction hair growth by 29 days, as determined by scores increasing from and then returning to zero (Supplementary Figure S8A). Dorsal skin was sampled and prepared for RNA analysis or antigen staining at multiple time points throughout the cycle.

An exhaustive investigation was not within the scope of this work; therefore, we identified a subset of candidate genes for experimental follow-up. Candidates were chosen by considering the significance of periodic expression and the t-statistic derived in the 2-population model. Additionally, we considered genes that had plausible, but not well defined connections to hair growth and regulation as determined by the literature. We chose Signal Transducer And Activator Of Transcription 5A (Stat5a), Fermitin Family Member 2 (Fermt2) and Vimentin (Vim) for candidates associated to the background population, as well as Ovo-Like 1 (Ovol1) and SMAD Family Member 6 (Smad6) for the expanding population. In the follicle dissection study of Rendl *et al.* [36], Stat5a and Ovol1 were also identified as signature genes for DP and MX cells, respectively; however, the other candidates were not linked to a specific cell type in the same study. The metrics relating to these candidate genes as well as others identified in this study are presented in Supplementary File S2. For candidate and control genes, we confirmed the expected relative expression profiles by qRT-PCR (Supplementary Figure S8B,C). Specifically, we confirmed that the relative expression of background candidates decreased during anagen onset and then increased after completion of anagen with maximums observed in or near the telogen phase, while the expanding population demonstrated a reciprocal profile (recall Figure 1, cluster 2 and 1 respectively).

**Figure 6:**
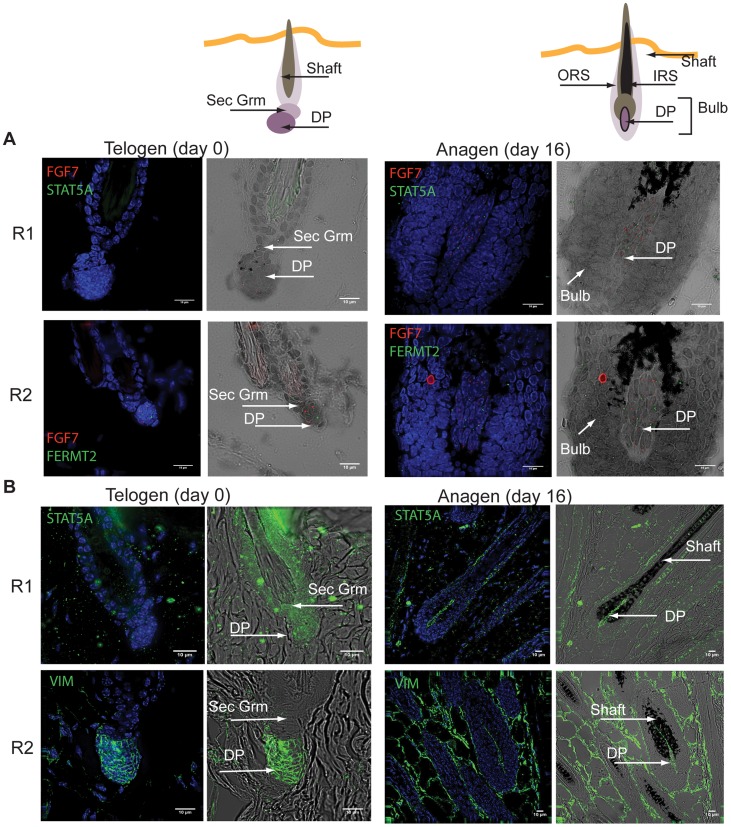
Localization of selected candidate genes from the predicted static background population markers. In Situ Hybridization (ISH; RNA) and immunofluorescence (protein) were performed on mouse skin sections taken from telogen (day0) or anagen (Day 16) phases of the hair cycle, determined by Supplementary Figure S8 A. DAPI was used as a counterstain for cell nuclei (blue). The expression of candidate genes for ISH is seen as bright foci (red and green) in specific cell types. Note comparisons to technical negative control and positive controls in Supplementary Figure S9. (**A**) ISH: Fgf7 (red) was used as a positive control marker, which has been reported to be expressed in DP cells [42]. R1 and 2 shows RNA expression for Stat5a and Fermt2 (green), respectively, which were predicted to be expressed in dermal papilla, or other background population during the telogen and anagen phase. (**B**) Protein staining by immunofluorescence: Due to technical issues with Fgf7 and Fermt2 antibody selection, morphology was used to determine localization and Vim was chosen as an alternate candidate marker. Vim was predicted to be expressed in dermal papilla or other background population during the telogen and anagen phases. R1 and 2 shows protein expression for Stat5a and Vim (green), respectively.

We investigated the localization of candidate gene products by various imaging techniques in samples corresponding to telogen taken before induction (day 0) and anagen taken 16 days after induction (day 16). Here, we recall that background candidates were determined to be markers for hair follicle cell populations that remain relatively stable throughout the hair cycle; these were also enriched with DP signature genes. Using ISH and fibroblast growth factor 7 (Fgf7) as a control marker [42], we confirmed that the signature gene Stat5a, also identified by the 2-population model, was expressed in DP cells (Figure 6A R1). We note that technical negative and positive controls for ISH can be seen in Supplementary Figure S9. Interestingly, ISH imaging produced similar expression results for background candidates in both telogen (day 0) and anagen phases (day 16) while qRT-PCR and microarray suggested notable differences in relative expression between these phases. These observations were explained by the 2-population model above, where increases in the expanding population decreases the relative size of the background population, resulting in notable expression changes for mixed cell population samples, such as the whole skin samples used in qRT-PCR. Although consistent with our expectations as described, for completeness we add that insensitivity in ISH imaging could provide another explanation for the observations. Furthermore, significant changes of Fgf7 expression have been detected in DP isolates [37], although under different conditions from the data considered here. We observed the same localization pattern in the novel candidate marker, Fermt2. Roles for Fermt2 have been proposed in regulation of the extra-cellular matrix, actin organization as well as cell-ECM focal adhesions [43]. It is also associated with *β*-catenin/TCF4 complex, and knockdown of Fermt2 leads to loss of *β*-catenin mediated transcription [44], ultimately affecting myogenic development. Other effectors of the *β*-catenin/TCF4 complex, such as Wnt, are known to be required for the hair inducing property of DP cells [45]. Background population proteins were also localized by immunofluorescence. The morphology, as determined by brightfield and DAPI staining, was used to identify DP localization. We observed localization of Stat5a protein to the DP in anagen samples; however, localization was much more difficult to assess in telogen samples (Figure 6B R1). Technical issues prevented antibody staining for Fermt2; therefore, we considered an alternate candidate, Vim, which was also associated to the background population. Immunofluorescence demonstrated Vim protein expression localized to the cytoplasm of DP cells in both telogen and anagen phases. We also observed Vim staining in the dermal sheath that surrounds the anagen bulb (Figure 6B R2). While Vim expression in the follicle has been reported [36], [46], [47], it is expressed in other dermal cells and it is thus not specific to the hair follicle. Its expression in both DP cells and in cells adjacent to the hair follicle, or macro-environment, emphasizes the importance of the use of whole skin in our study. For example, Plikus *et al.* [48], [49] demonstrated that the macro-environment can be the source of paracrine signals that influence the hair cycle.

**Figure 7:**
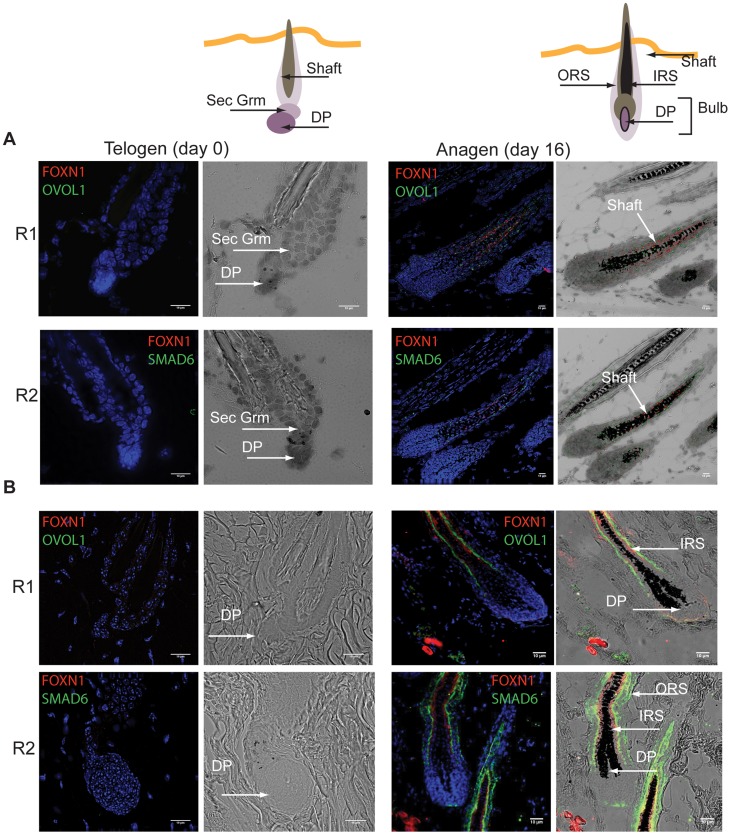
Localization of selected candidate genes from the predicted anagen expanding population. In Situ Hybridization (ISH; RNA) and immunofluorescence (protein) were performed on mouse skin sections taken from telogen (day0) or anagen (Day 16) phases of the hair cycle, determined by Supplementary Figure S8A. DAPI was used as a counterstain for cell nuclei (blue). The expression of candidate genes for ISH is seen as bright foci (red and green) in specific cell types. Note comparisons to technical negative control and positive controls in Supplementary Figure S9. Foxn1 (red) was identified as a candidate matrix derived cell marker and was used here as a positive control for localization to matrix cells [50]. (**A**) ISH: R1 and 2 shows RNA expression for Ovol1 and Smad6 (green), respectively, which were predicted to be expressed in follicle cell populations that expand during the anagen phase. No expression was observed during the telogen phase (Day 0). (**B**) Protein staining by immunofluorescence: R1 and 2 shows RNA expression for Ovol1 and Smad6 (green), respectively. Again, no expression was observed during the telogen phase (Day 0).

Imaging results also confirmed candidate markers for the expanding population. Here we recall that the expanding population candidates were determined to be markers for cells whose relative population size increases during anagen, followed by a sharp decline in catagen (Figure 4); these were also enriched for MX cell genes. Using Forkhead box protein N1 (Foxn1) as a positive control for MX cells [50], note: Foxn1 was also identified in the current study as a candidate marker for the expanding population), we observed mRNA expression of the signature gene Ovol1 in the proliferating cell populations of the hair shaft in anagen samples, as described in the literature [36], [50]–[52], Figure 7 R1). We also observed the same expression pattern and localization to the hair shaft for the novel candidate marker Smad6 (Figure 7A R2, day 16). Further evidence of an association between these candidate markers and the expanding population was demonstrated by a general lack of staining in telogen samples. The 2-population model attributes this lack of expression to the absence of the expanding cells in the telogen phase (Figure 4). Additionally, immunofluorescence confirmed the localization of Ovol1 and Smad6 protein near the MX cell marker Foxn1 (Figure 7B). Matching the pattern of Foxn1 expression at day 16, ovol1 and Smad6 stained the epithelial, matrix-derived inner root sheath cells and the upper part of the matrix cells that surround the hair bulb while Smad6 was also detected in the epithelial outer root sheath. Although their general expression was observed, Vimentin and Smad6 were not identified as signature genes for DP and matrix cells respectively by Rendl et al [36]. The results here (Figures 6 and 7) demonstrate their expression in distinct compartments relating to follicle epithelial (Smad6) and background mesenchymal (Vim) cells. The staining results and the 2-population model support the hypothesis that the computationally identified expanding population was associated to follicle epithelial cells. Currently there is no direct evidence of Smad6 in hair cycle regulation; however, Smad6 is a well-known negative regulator of the Tgf*β* signaling pathway [53], [54]. Given its role in regulating Tgf*β* signaling, as well as its proximity to MX cell markers, Smad6 may be an important candidate for future study in hair cycle regulation. We also noted the periodic expression of bone morphogenetic protein (BMP) genes, which have been documented as important regulators of skin and hair development. Four BMP genes showed periodic expression as LFOs: BMP 1 was found in cluster 1 (dermal papilla-associated) while BMPs 2K, 8a, and 7 were expressed in cluster 2 (matrix-associated). Other BMPs such as 2 and 4 were present in the original data, but the expression data contained too much variability to survive the FDR cutoff. The complete lists of LFO genes that matched the background and expanding population clusters, along with statistical metrics, is in Supplementary File S3.

### Identification of negative feedback targets

The coupled oscillator model suggests that out-of-phase clustering is maintained by positive and negative coupling. The two population model indicates that these clusters are associated to specific cell populations. Taken together this is similar to negative feedback, for example the expanding population may drive the background population to produce an inhibitory signal, such as apoptosis, that in turn depletes the expanding population. However, if the background population is static, how is it contributing to such a control loop? For example, when the expanding population is relatively high, one would expect an increase in the expression of the inhibitory genes from the background to drive down the expanding population. One reasonable possibility is that expression changes were occurring within the background population. On average we found that the assumption of static intracellular expression was reasonable enough to estimate population dynamics (recall Figure 4); however, many individual expression profiles were poorly described by this assumption (recall Figure 5A). It is possible that these poorly described genes were undergoing intracellular changes, and could be responsible for the physical communication of inhibitory signals from the static background to the expanding population.

We investigated the possibility that inhibitory signaling genes may be in the DP enriched group identified as LFO cluster 2, but not well described by the static intracellular expression model. We expect such signaling genes to display an increased expression 14 to 16 days after morphogenesis, near the on-set of catagen and before the sharp decline in the expanding population (Figure 4). Using this criterion, we identified 88 expression signals (relating to 74 unique genes; see Supplementary File S4). We observed that these expression signals, on average, are consistent with population driven changes until near catagen on-set, where they begin to increase more than what was explained by static intracellular expression assumed in the 2-population model (Supplementary Figure S10). Of these genes, 50 were annotated as extracellular genes which yields and enrichment p-value of 7.46E-18, improved enrichment over cluster 2 with a p-value of 3.63E-8. For a full list of significant enrichment categories see Supplementary File S5. Interestingly, this relatively short list includes Tgf*β*2, which is currently thought to be one of the signaling molecules produced in DP cells to initiate apoptosis in hair epithelial cells at catagen on-set [7].

Given the observed expression signal, membership in DP enriched cluster 2, high enrichment for extracellular genes and inclusion of Tgf*β*2, this list may contain potential targets for molecules that communicate an inhibitory signal from the DP to proliferating hair epithelial cells, closing a negative feedback loop. Obviously further experiments will be required to test this hypothesis; however, it does provide a starting point for future validation of the conclusions drawn above and, perhaps, even those identified in the model of Al-Nuaimi *et al.* [10].

### General limitations to overall study

Although our approach provides novel insights and genes associated to the hair follicle, we also recognize that there are several limitations to this study. We studied microarray-derived RNA expression data from developing mouse skin that included non-periodic as well as periodic gene expression patterns. Due to the cyclic nature of the hair cycle, we chose to focus our study on the latter. We emphasize that our study would overlook important regulators of the hair cycle if they were not periodically expressed. Next, we only considered a single time course, experimental study (Lin et al, 2009), which obviously limits the data and conditions available to us (sparse sampling, limited technical replicate measurements and inclusion of only early cycles), and could lead to some important genes and cycle dynamics being excluded from further analysis. Furthermore, biological and technical variation, along with typical tradeoffs in sensitivity versus specificity associated with parameter selection, such as p-value thresholds, will further limit statistical detection of important mRNAs or expression patterns. Due to concerns of batch effects, we did not choose to combine additional datasets from other experimental studies to offset these issues. Instead, we chose to limit the scope of our investigation to describing a specific, but prevalent, dynamic pattern observed in the data. Again, by limiting the scope in this manner, it is likely that some important hair cycle regulators were overlooked. For example, BMP 2 and 4 have been shown to influence anagen initiation [48], [49], but due to sample variability these patterns fell outside the range of detection for this study. However, our investigation did encompass over 3,000 unique genes, where follow-up dynamic, enrichment and experimental analyses all suggested a possible role in the hair follicle and cycle dynamics.

Study design also limited us to time course over a single cycle of follicle synchronized hair growth. We were not able to test if the identified expression patterns, specifically synchronized out-of-phase gene expression, continued for additional cycles. This is a typical experimental limitation due to the loss of follicle synchronization as animals mature, at latter stages of hair growth. This is a different concept from the synchronization describing gene expression patterns. One might still expect that similar gene expression patterns within individual follicles, and the surrounding microenvironment, continue with additional cycles; however, without single follicle tracking, we cannot confirm this. Furthermore, our dynamic coupled oscillator model would never predict such follicle-level de-synchronization, as we did not include any mechanisms for cycle variability nor did we include the concept of individual follicles. Accounting for stochastic variation and spatially modeling individual follicles that are themselves coupled, represents an additional level of complexity that may more accurately model the rich dynamics of the hair system, but was not considered in this study.

Finally, we modeled gene expression from whole skin, since isolation of hair follicles prior to gene expression profiling is resource intensive and was beyond the scope of our work. In doing so, we relied on the 2-population model, cell type specific enrichment (based on experimentally purified cell populations [36], [37]) and experimental imaging to make associations between computationally derived gene groupings and distinct biological populations. While these results were very encouraging, we would like to emphasizes that the computationally derived populations do not represent a specific, predefined cell type. Supplementary Figure S7A shows that the majority of the signature genes were not identified, and the populations contained signature genes from multiple cell types. However, we do note that even experimentally derived gene signatures also demonstrate overlap (see Supplementary Figure S7B). Furthermore, it likely that cell types not investigated by enrichment also contributed to the estimated populations, such as endothelial cells involved in capillary network remodeling, adipocytes or immune cells that may have active roles in hair growth. Ultimately, we identified many associations between the computationally derived gene groupings and distinct, hair cycle relevant cell populations, but we cannot exclude that gene expression unrelated to the hair follicle or hair growth may have contributed to both false positives and negatives.

### Conclusion

**Figure 8:**
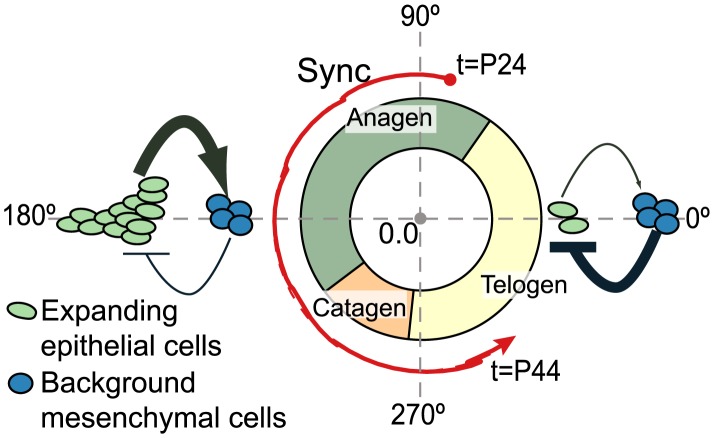
Summary schematic of the hair oscillator. Synchronization (red line, corresponds to Figure 2B) is shown to follow the temporal trajectory of the hair follicle. Rough predictions of relative size for expanding matrix cell and background dermal papillae populations are indicated at specific times in the cycle by green and blue ovals, respectively (similar to figure 4). The arrow and bar indicate positive and negative coupling to the mean field, respectively. The symbol is shown in bold when corresponding genes reach there maximum as estimated in Figure 1C.

In this study, we focused on potentially periodic gene expression patterns in whole skin that changed on the same time-scale as cyclical hair growth. We identified two distinct clusters consistent with synchronized, out-of-phase gene expression (Figure 1 and 2). Through nonlinear-dynamic analysis, we proved that a simple, coupled oscillator model was mathematically sufficient to recapitulate this observed synchronization, and that the coupling scheme involves both positive and negative coupling (Figure 3A). We go on to show that these clusters can be associated with either static or expanding cell populations (Figures 4 and 5), and that the size of the expanding population, determined by gene expression data, was consistent with the population dynamics of follicle epithelial cells (Figure 4). Follow-up experimental and enrichment analyses indicated that the corresponding genes (provided as Supplementary Information File S3) were strongly associated with biologically distinct cell-types, such as MX or DP cells (Figures 6 and 7, Supplementary Table S1). Taken together, these results were consistent with regulatory mechanisms involving negative feedback from background mesenchymal cells to the expanding epithelial cells (see summary Fig 8). Finally, we identified a subset of genes that could potentially communicate the inhibitory signal to the follicle (provided as Supplementary Information File S5). Other aspects of the study provided interesting, but speculative, insights on possible alternative hair cycle states that are similar to those of miniaturized hair follicles (Figure 3B).

The model of hair cycle presented here suggests some role for proliferating follicle epithelial cells to be regulated by a systems-level inhibitory response, likely to emanate from the DP. Conceptually, regulated apoptosis could be one way in which a large number of genes from the same general population of cells are inhibited by a second population. This mechanism has also been explored in a kinetic model of hair cycle which shows that negative feedback via DP regulated apoptosis is sufficient to account for the cyclical nature of hair growth [10]. Because mRNA expression data underlies the model presented here, we were able to advance this idea a step further and identify candidate signaling proteins based on the dynamics of the gene expression. Although experimental confirmation of these candidates was beyond the scope of the investigation, we note that the list was highly enriched for extracellular proteins and with only 74 genes we were still able to identify Tgf*β*2 as a possible candidate. For clarity, we emphasize here that negative coupling is not the only aspect of our model, which also includes intrinsic oscillations and positive coupling. In fact, our model predicts that reduction of the DP associated oscillators actually results in a shortened cycle (see Figure 3B). Furthermore, the true physical mechanisms underlying the hair cycle are likely far more complex, and studies show that anagen length and hair size actually decrease upon depletion of DP cells [55]. Both our computational and existing experimental results suggest that inhibitory regulation of MX derived cells cannot describe all aspects of the hair cycle; however, it is likely to play an important role, with one possibility being regulated apoptosis [7], [56], and the genes we have identified here could help guide follow-up experiments.

Finally, the most intriguing aspect of this study was the predicted proximity of the hair system to a critical phase transition (Figure 3B). In the observed dynamics the hair system was in a stable 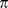-state, in which the proportion of positive and negative oscillators may vary, or at least increase, without affecting the overall period. However, a decrease in this proportion, for example a reduction in positively coupled oscillators, would throw the system in to a travelling wave state, where the two clusters drive each other into higher frequency oscillations. If this behaviour can be validated, it would have important biological implications. Biologically the model suggests that there is a systems-level regulation within and between genes related to follicle epithelial and background mesenchymal cell populations, which represents a balance of negative and positive driving forces. Loss of regulation of the DP population, for example, would mean a decrease in negative forces acting on the epithelial population and would throw the system into a fundamentally different state. This new state would be characterized by a drastically reduced period of expression, similar to hair miniaturization resulting from androgenetic alopecia. Unfortunately, testing of this hypothesis could prove difficult. Removal of several genes via genetic knockout would have consequences not accounted for here; however, inhibition of the physical signaling between the DP and MX derived populations may be more tractable. Excitingly, a recent study by Rompolas *et al.* [57] introduced new methodology to explore such interactions of the hair follicle in live mice using laser ablation of DP cells. The approach not only allowed for the elimination of a specific cell population, but also removed technical complications associated with follicle synchronization, as individual follicles could be monitored over time. It would be of future interest to see if this methodology could be modified to properly test the predictions presented herein.

To build on the insights of the present framework, we can offer several additional directions for future work. For example, there are several possible advancements to the coupled oscillator model, including: analytically solving the existing coupling scheme for excitable elements (similar to [58]) opposed to oscillators would better capture the pulsing behavior of gene expression and hair growth; integrating noise, known to have a major impact of synchronization [59], could help capture both expression and cycle variability; and coupling together multiple coupled systems could capture associations and variation between follicles. Experimentally, new time course data could identify new behaviors. Performing a single extensive time course from morphology to end of second round of hair growth would capture anticipated transients and determine a proper time scale for synchronization. Increasing the sampling frequency could identify high frequency oscillators and perhaps provide a means to couple circadian rhythm to the current system. Given that we systematically identified two clusters from whole skin data, a beneficial advancement would be to directly collect data from follicle specific cell types, such as MX and DP cells. Producing a time course using purification techniques similar to Rendl *et al.* would be the most direct way to prevent confounding expression signals from non-relevant cell types and provide a specific interpretation of modeled populations. In our experience, any additional time courses would need to have, at a minimum, a sampling rate 3 times that of the desired frequency for identification. However, we would strongly suggest doubling the number of points in the time course and including 3 replicates at each point for statistical and modeling considerations. Our hope is, that by incremental advancement, the framework provided here can be used to bridge the gap between high-throughput measurement data and systems-level properties of hair cycling.

## Methods

### Ethics statement

This study was performed in strict accordance US Animal Welfare Regulations at an AAALAC accredited site. The research protocol was approved by the Institutional Animal Care and Use Committee of Procter & Gamble. Every effort was made to minimize suffering of all animal subjects.

### Data acquisition

We employed data originally generated in Lin *et al.* [11]. The authors profiled both second, naturally synchronized and depletion-induced hair cycles. Samples were collected from the upper-mid region of C57Bl/6 mice and analyzed using Affymetrix Mouse Genome 430 2.0. For additional experimental details please refer to the original article.

The raw intensity data was collected from the NCBI Gene Expression Omnibus as accession number GSE11186. The data was uploaded in CEL file format and preprocessed for quality control. Sample GSM281802 was removed based on suspected RNA degradation, a mean correlation coefficient less than 0.95, multiple outliers determined by the MA plot, and high background error and variation determined by RMA. The remaining samples were summarized and normalized using the RMA function from the Bioconductor ‘affy’ library in R, applying quantile normalization and RMA background correction from affy version 1.1. A log base 2 transform was applied to the expression data for all subsequent analysis except for the 2-population mixture model. An R script containing the general QC and RMA methods used can be found in the Supplementary File S6.

The two experimental conditions corresponding to the natural and induced hair cycles, were combined into a single time course as suggested by the original authors. The five sampled time points for the induced cycle, {3,5,8,12,17} days, were mapped to time points in the natural cycle, {24,25,27,29,37} days, based on the morphology of skin sections. Hair morphogenesis during synchronous growth was established by histologic criteria [11]. Combining samples with similar morphologies, and therefore in similar hair cycle phases, we expect to limit the variability of gene expression that is associated to the hair cycle phenotype.

### Visualization of expression data

We visualized the expression data using standard heat maps. Multiple values at a given time point were averaged. For visualizing expression values of different scales in a single image it was necessary to normalize the data. Two different normalization schemes were used. Figure 1 focuses on relative levels of periodically expressed genes, here we used a 0–1 normalization: 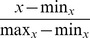. The associated time scale is ‘days postnatal’ and corresponds to the natural second cycle; the induced cycle was matched to time points as discussed above. In Figure 5 and the 2-population mixture model, we applied a fraction of max normalization, 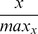, to capture information of relative fold change in expression. The associated time scale was days from initiation to emphasize differences in the natural and induced cycle, which is assumed to begin after morphogenesis, which is ≈23 days, and depletion, respectively. The normalization schemes described here were for visualization purposes, and were not used in any statistical analysis. For all time series, we present a color bar to roughly indicate the corresponding phase of the hair cycle, the timing for the color bar was taken from [11].

### Periodic identification

#### Fourier Series Decomposition of expression signals

To identify periodic signals in the mRNA expression data, we applied a robust regression method described in the literature [29]. This method allowed us to properly account for non-uniformly sampled time points, handle outliers in noisy expression data and estimate the Fourier series decomposition of the signal. The method attempts to estimate a frequency representation of the discrete signal, equivalent to a discrete Fourier Series Decomposition (FSD),
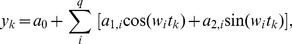
(4)by fitting the coefficients 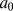, 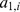 and 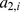 using robust linear regression over 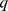 pre-selected frequencies, 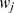. We applied an M-estimator with Tukey's biweight for the robust linear regression as suggested by the literature [29], [60]. Given the coefficients, we estimated the power spectrum defined as
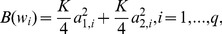
(5)where 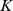 is the total number of discrete points that make up the signal. The power spectrum was then used to calculate Fisher's g-statistic [61]

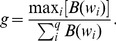
(6)The g-statistic summarizes the relative power of the principal periodic component (PPC), the strongest contributing frequency of the signal. We also estimated the goodness-of-fit using the standard coefficient of determination, *r* ^2^, (see Supplementary Figure S1) and the mean absolute deviation (see Supplementary Figure S11) for both the FSD and the PPC. Python code for estimating the FSD and calculating the g-statistic can be found in the Supplementary File S6 (at time of submission updates were maintained at https://github.com/Rtasseff/robustFourierSeries).

#### Estimation of statistical significance of periodicity in expression signals

A permutation strategy was used to find the p-value for the g-statistic. To improve p-value estimation, we applied a Generalized Pareto Distribution (GPD) to the tail of the permutation distribution when possible as described in the literature [62]. In some cases this can greatly reduce the number of permutations needed to approximate low p-values. We used one thousand to one hundred thousand permutations as needed to apply the GPD or find ten excedents to the g-statistic for calculation of the p-values. Finally, the false discovery rate was calculated over all probesets and a threshold of 0.10 was applied [63].

The complete periodic identification procedure was performed on each of the normalized expression signals. No pre-filtering or pre-selection of probesets was applied. We note that the selection of frequencies is not obvious for non-uniformly spaced time points; however, false positives due to improper frequency selection are mitigated by subsequent p-value calculations. We did find that the inclusion of very high frequencies, near or greater than 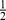 the minimum sampling rate, or very low frequencies, corresponding to periods much greater than the range of the time course, tended to ‘mask-out’ other frequencies in the signal when calculating the g-statistic (EQ 6), resulting in an increase of false negatives. This was due to choosing a frequency for the g-statistic before, and independently from, the p-value estimation. The frequencies investigated along with the number of probesets identified are shown in Supplementary Figure S1. Python module for the application of the GPD can be found in the Supplementary File S6 (at time of submission updates were maintained at https://github.com/Rtasseff/gpdPerm).

### Characterization of periodic signals as oscillators

#### Calculation of instantaneous state variables for individual oscillators

To describe the expression dynamics in terms of oscillators, we calculated the instantaneous phase and frequency. If we imagine an oscillator as a point revolving around the unit circle in the complex plane, then we can describe its trajectory by finding its phase (or angle) and frequency (or rate of change). Here, trajectories are a result of the microarray time course data for samples of mouse skin, and therefore represent averages over this tissue. Given an analytical, or continuous, representation of the signal, we can use common methods applied in signal processing to calculate these properties [64]–[66]. Above, we estimated FSD of the periodic expression signal which can be further simplified by only considering the term associated with the PPC. Both the FSD and PPC provide us with an analytical representation of the signal, and Supplementary Figure S1 and 10 shows that both are reasonable fits to the data in terms of the coefficient of deviation and the median absolute deviation. Next, we move to the complex plane by calculating the so called analytic signal, 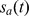, defined as:
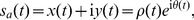
(7)where the real part, 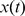 is the original signal at time 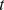 and the imaginary part, 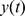, is the Hilbert transform of the signal, i is the imaginary unit, 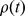 and 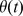 are the instantaneous amplitude and phase, respectively. For 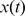 we can use the discrete expression values (shifted by 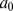 from EQ 4) or one of the continuous approximations FSD or PPC (also determined by EQ 4). To solve for 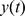, we can simply replace 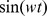 with 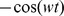 and 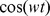 with 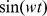 in the FSD or PPC from EQ 4. From this we can easily see that 
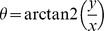
. Where 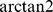 is the standard variation of the arc tan function that provides sign information. Finally, we can calculate the instantaneous frequency by taking the derivative:
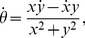
(8)where the dot indicates differentiation with respect to time. We note that this formulation avoids discontinuities at the 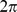 intervals. We also note that the instantaneous amplitude can also be calculated, but will not be used in subsequent analysis. Python code for calculating oscillator properties can be found in the variables module in the Supplementary File S6 (at time of submission updates were maintained at https://github.com/Rtasseff/oscillator).

#### Calculation of complex order parameters for a system of oscillators

We calculated a set of complex order parameters to quantify the collective behavior of the system. In oscillatory systems it is common to use the order parameters 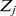 defined as:
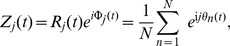
(9)where 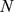 is the number of oscillators (gene expression trajectories identified as periodic) and 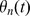 is the instantaneous phase or the position of oscillator 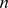 on the unit circle at time 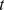 [31], [32] calculated using EQ 7. Here the subscript 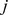 on the left hand side indicates which order parameter is being calculated, higher values of 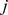 consider higher harmonics of synchronization in the system. Note that EQ 9 is identical to EQ 1 presented in the Results and Discussion, and was included here only to maintain the continuity of the method descriptions. Figure 2A shows the magnitude of the first and second order parameter calculated over the low frequency oscillators (period corresponding to 31 days) and randomized signals. We computed random signals by adding in a phase shift chosen uniformly at random over 0–2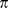 for all oscillators. We note that the magnitudes are similar when calculated over all signals found to be periodic and not limited to the low frequency oscillators (Supplementary Figure S12). In this analysis, we also considered individual order parameters for each cluster. This was done by doing the sum in equation 9 over the oscillators associated to a specific cluster. We used lowercase letters, for example 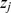, to distinguish these from the global order parameters. Figure 2B shows the first order parameter for the full system as well as the two clusters. Python code for calculating order parameters can be found in the metrics module in the Supplementary File S6 (at time of submission updates were maintained at https://github.com/Rtasseff/oscillator).

### Coupled oscillator model

We employed a mathematical description of coupled oscillators to study general features of the synchronization observed in the hair cycle data. We considered a modified version of the simple Kuramoto model [16] suggested by Hong and Strogatz [33]. Hong and Strogatz show that a two group model, one positive and one negative, is sufficient to spontaneously produce two out-of-phase clusters. Oscillators which are positively or negatively coupled will be drawn towards or pushed away from other oscillators on the unit circle, respectively. After some simplification and incorporation of EQ 9 the governing equations reduce to
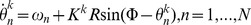
(10)where 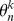 is the phase of the 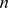
th oscillator, calculated by EQ 7, which is assigned to group 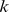, 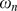 is the natural or intrinsic frequency for 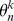, 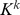 is the coupling constant for group 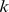, 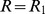 and 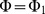 from EQ 9 where the subscript is dropped for simplicity. The dot denotes change with respect to time, and 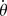 can be calculated using EQ 8. Recall that oscillators represent probesets with expression patterns identified as periodic. Here we assume two groups 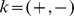 where 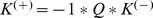 and 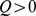, 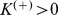, 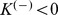. Introducing these two groups to EQ 10 we have
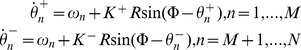
(11)where 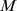 is the number of positively coupled oscillators. We note that EQ 11 is identical to EQ 2 presented in the Results and Discussion, and was included here only to maintain the continuity of the method descriptions. We also reemphasize several simplifications in this formulation. First, the model is a mean field approximation in which each individual oscillator is connected to all other oscillators through the order parameter, 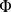. This is derived from an assumed all to all connectivity, which is obviously not expected in a gene network; however, the mean field approximation works well if the effective coupling on the oscillators (or genes) is well described by an average of the individual couplings. Such models have successfully described high level properties of many large, complex systems including statistical mechanics (overview of several models [17]), economics [18], [19] and even social networks [20]. Second, we note that the variables here are considered independent, for example the model assumes that the proportion of oscillators can be varied without affecting other independent variables, such as the ‘intrinsic’ oscillations, 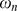. However, in reality removal of genes from the system will have an impact not captured in the model, such as an alteration or even cessation of the assumed ‘intrinsic’ oscillations. Finally, we emphasize that the coupling describes oscillator interactions and not necessarily the underlying driving force for oscillation, which is typically attributed to 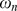. With this level of abstraction and simplification it was not possible to describe most of the details of the hair cycle including mechanistic molecular connectivity; however, we were able to describe more general aspects of the system such as a stable, synchronized state.

To solve the system, we follow the original paper [33], and summarize the process here for the reader's convenience. We can reduce the model to a low dimensional system in terms of the first order parameters for each group. First, we consider a system where 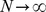, we validate the use of this assumption later. Second, we assume the 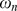 were randomly distributed via a Lorentzian probability distribution 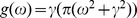. Importantly, we note that use of a single frequency, opposed to a distribution, will not recapitulate the distributed phases observed in Figure 1C [67]. Here, we have moved to a rotating frame such that the mean of 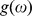 is zero; in our system this was done by subtracting the principal periodic component, 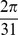 radians per day. Finally, we can apply the ansatz of Ott and Antonsen [68] which yields
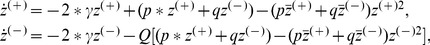
(12)where 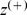 and 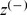 are the first order parameters (similar to EQ 9 with 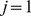) for the two oscillator groups related to positive and negative coupling, respectively; 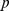 is the proportion of positively coupled oscillators and 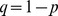. The bar denotes complex conjugate. We note that EQ 12 is identical to EQ 3 presented in the Results and Discussion, and was included here only to maintain the continuity of the method descriptions. Using EQ 12 we can solve for the critical value of 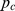 such that only the incoherent state is stable when 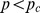 and is, therefore, the lower bound for observing synchronization. We can also estimate other bifurcation points of the system, 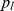 and 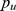. For more details see [33].

We solved for various properties of the hair cycle system using EQ 12 and the oscillator state variables solved for above. We assumed the observed period of the hair cycle system was at a quasi-steady-state, where the magnitude of the first order parameter is constant and the rate of change of the phase is also constant. This was demonstrated by 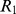 observed in Figure 2A. Given the quasi-stead-state, we solved EQ 12 in a rotating frame described above, allowing us to set the left hand side to zero. We considered two possible configurations of assigning clusters (from Figure 1C) to positive or negative coupling groups. After coupling assignment, we calculated 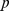, 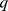, 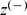 and 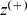 from the data and solved EQ 12 for the unknown parameters 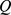 and 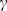. Then 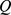 and 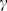 were used to solve for 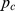. We note that EQ 12 was solved by letting 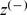 and 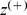 lie on the real axis so they were equivalent to 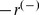 and 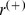, respectively. This assignment can be done, without loss of generality, for a quasi-steady-state, out-of-phase system. For the configuration with (clust1) = (+) and (clust2) = (−), we found that the system was unstable (Figure 3A purple dashed line). We calculated 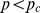 and 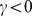 which is not physically realizable. However, we found the opposite configuration, with (clust1) = (−) and (clust2) = (+), to be a stable solution with 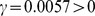 radians per day and 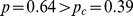 (Figure 3A red solid line, recall actual data in Figure 2A red, see Supplementary File S2 for a simulation describing individual oscillators with these properties).

The bifurcation diagram was solved numerically (Figure 2B). We found the long time solutions to the system of ordinary differential equation (EQ 12) for various values of 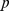 while holding all other variables constant. We found 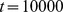 to be sufficient. We verified the assumption of 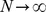 by simulating the low dimensional system, EQ 12, and comparing that to simulations of the high dimensional, EQ 10, with 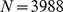 (Supplementary Figure S13). Given noise, due to initial configurations, associated with the finite, high dimensional system, 100 iterations were calculated and the mean and standard deviation were reported. The two representations are sufficiently similar and have nearly identical steady-states. All numerical simulations were performed in Matlab [69] using ‘ode45’. All Matlab scripts necessary to solve for model variables and reproduce simulations found in figures and movies are available in the folder ‘meanField’ in Supplementary File S6.

### Estimating relative size and expression of two cell populations

Observations of two distinct gene expression clusters motivated us to explore possible relationships to different cell populations within the hair follicle. We considered the scenario in which observed expression changes are due to changes in relative cell population size as opposed to intracellular changes. In Supplementary Figure S3, we showed a simple example in which 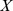 and 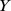 had a high and low concentration in pop 1, respectively, and reciprocal concentrations in pop 2. While varying the size of pop 1, holding the pop 2 size constant and holding all internal concentration level constant, we can see that the observed concentration of 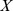 and 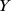 change, and do so in an out-of-phase manner. To explore this in our system we wished to reverse the process and estimate the relative sizes and intracellular expressions given the observed mixed expression. To achieve this we applied *in silico* microdissection [35].

Briefly, *in silico* microdissection works by applying a simple linear model of mixed samples
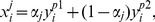
(13)where 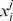 is the observed expression of gene 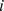 in mixed sample 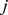; 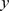 indicates the intracellular expression in populations 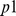 and 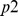; and 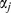 is the cell fraction of 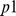 in mixed sample 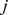. Given the cell fraction, 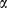, for each sample we can solve EQ 13 for the internal concentration in the two populations. In this situation each gene 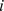 is an independent problem, each solved via simple linear regression over all mixed samples 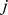. This is an overdetermined system if the number of populations considered is less then the number of samples. We can also recast the problem to solve for 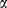 given the internal concentration for each population. In this situation each mixed sample 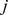 is now an independent problem, each a constrained linear problem over all genes 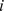. Here 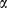 is constrained between 0 and 1, the problem is convex and can be solved efficiently. An iterative process, similar to expectation-maximization, can be used to solve for both 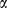 and the *y*'s simultaneously.

We consider a model of an expanding cell population mixed with a constant background population. We treated the hair cycle expression chips as independent mixed samples each with possibly different cell fractions. No information of cycle type or time was needed, nor any strategy for combining samples as in the previous periodic identification. For later comparisons of the induced and natural cycle, we set the time relative to cycle initiation, which we assumed to be after morphogenesis (postnatal day 23) or after depletion. This time scale was only used for graphical representation, and was not used in any calculations. A linearly increasing function from 0 to 1 was used as the initial conditions for 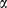, the cell fraction of the expanding population. We also included the expression of all 45k+ probesets without the log2 transform, as suggested in [35], all other preprocessing was the same. Given this, over determined, model we implemented the above iteration strategy to solve for 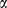 and the relative size (Figure 4) and the internal expression (Figure 5C). We note here that it is not reasonable to expect all intracellular expression to remain constant over the whole hair cycle; however, if the relative population change is large, as seen here, compared to the intracellular expression change for many genes then it is a reasonable assumption.

While calculating the internal expression for the two populations, we also estimated the corresponding standard error using common methods associated with linear regression. The standard error was used to produce a t-statistic and p-value for each probeset, which indicated the extent to which a gene was differently expressed between the two populations (Figure 5B). The probesets for the LFO subgroup with a t-statistic above a 0.10 false discovery rate were assigned to the population in which they were predicted to have higher expression. We note that nearly all of the LFOs met the statistical requirements, 3975 out of 3988. This was equivalent to separating the probesets into two groups based on the estimated t-statistic, and was found to be equivalent to separation of LFOs by phase (Supplementary Figure S6).

We also considered a computational negative control. In the above analysis, we inherently assumed that expression is related to time, after morphogenesis or after depletion. Our population analysis allowed us to then associate expression to relative population size, and therefore, plot relative population size as a function of time. Here we considered a negative control, that expression is random with respect to time, and not related to hair cycle. To achieve this, we randomly permuted (or shuffled) the time courses for each probeset. For a proper comparison the depletion and naturally induced time courses were not intermixed, and kept separate. After permutation, we employed the exact same analysis and plotting procedure as above (used to produce Fig. 4 and 5). The results are shown in Supplementary Figures S4 and S5. In our negative control, we could see that there was no indication of expansion and depletion corresponding with anagen and catagen, respectively (Supplementary Figure S4). Furthermore, we did not observe improved statistics, as in coefficient of determination or the t-statistic (Supplementary Figure S5). We considered this sufficient evidence that the negative control produced only random population changes with respect to time, as expected.

All code for estimating the two populations was written in Matlab and implemented by the scheme discussed in [35]. The constraint linear problem was solved using Matlab Optimization Toolbox function ‘lsqlin’ with default options. Standard errors were estimated using the Matlab Statistics Toolbox function ‘regstats’. Matlab scripts for running all analysis described above and for generating the data in associated figures are available in the ‘2pop’ folder in Supplementary File S6.

### Functional, hair related, and cell type enrichment analysis

We used the online tool DAVID 6.7 to perform basic enrichment analysis [70]. We used the full mouse genome as the background gene set. For biological process enrichment, we used the Gene Ontology annotations under ‘GOTERM_BP_FAT’ and for pathway enrichment, we used KEGG annotations under ‘KEGG_PATHWAY’. The enrichment was done using different target sets indicated in the main text.

We used the Normalized Google Distance (NGD) to estimate enrichment of genes related to hair. The NGD is a semantic similarity measure [71], which for two terms 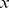 and 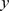 is defined as
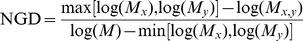
(14)where 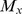 and 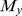 are the numbers of pages the terms 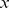 and 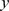 are found in, respectively, 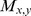 is the number of co-occurrences and 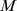 is the total number of pages considered. We note that the more frequent 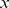 and 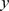 co-occur the lower NGD will be, and that if 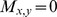 then NGD = inf. Here we applied the search to all abstracts in the NCBI PubMed database. We calculated the NGD between the term ‘hair’ and all mouse gene symbols. All genes with an NGD to ‘hair’ of less than 1.0 were used as the final set of Hair related genes. We applied a standard enrichment test using a hypergeometric distribution. The target set was the list of all periodic genes and the background set was the full mouse genome.

The threshold of 1.0 was chosen as it is the NGD of the expected value for independent or unrelated terms. Briefly, given a set number of occurrences for a term 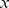 then the probability of finding term 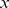 in 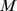 pages is 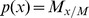. Assuming that two terms, 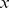 and 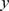 are independent, we have 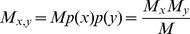. Plugging these values into EQ 14 we find that NGD = 1.

A cell type enrichment analysis was used to link model populations to specific cell types. Two existing studies, Rendl *et al.* [36] and Greco *et al.* [37], dissected hair follicles into specific, predefined cell types relating the the hair follicle: dermal papilla, melanocytes, matrix cells and outer root sheath cells from [36] and Bulge cells from [37]. Using mRNA microarray data the studies defined gene signatures for each population as sets of probesets and corresponding genes with expression nearly exclusive to a particular cell type. Using these signatures to annotate probesets with a particular cell type, we applied standard enrichment tests using a hypergeometric distribution. The target set was the list of genes determined to be in the expanding or background population (seen in Figure 5C) and the background set was the full mouse genome.

### Induction of hair cycle in C57Bl/6 mice

Male mice, C57Bl/6 (Charles River Laboratories, Portage, MI) at 62-66 days of age, in the telogen phase of the hair cycle [5] are shaved in the dorsal area (area of 1.5 inches×2 inches) followed by treatment with Nair (Church & Dwight Co.) to the same area for 1 hour before washing off to initiate the hair cycle. Nair depletion induces a similar response as wax by damaging the hair shaft to start a homogenous re-entry into anagen [72]. Mice were collected at various timepoints after induction treatment.

### mRNA localization by in situ hybridization (ISH)

ISH was performed using QuantiGene ViewRNA protocols (Affymetrix, Santa Clara, CA). Five *µ*m formalin fixed paraffin embedded (FFPE) sections were cut, fixed in 10% formaldehyde overnight at room temperature (RT) and digested with proteinase K (Affymetrix, Santa Clara, CA). Sections were hybridized for 3 hours at 40°C with custom designed QuantiGene ViewRNA probes against specific target genes and the positive control genes used were Fgf7 for dermal papilla cells and Foxn1 for matrix cells (Affymetrix, Santa Clara, CA).

Bound probes were then amplified per protocol from Affymetrix using PreAmp and Amp molecules. Multiple Label Probe oligonucleotides conjugated to alkaline phosphatase (LP-AP Type 1) were then added and Fast Red Substrate was used to produce signal (red dots, Cy3 fluorescence). For two color assays, an LP-AP type 6 probe was used with Fast Blue substrate (blue dots, Cy5 fluorescence) followed by LP-AP type 1 probe with Fast Red Substrate (red dots, Cy3 fluorescence) to produce a dual colorimetric and fluorescent signal. The probes sets used for ISH are described in Table S2. Slides were then counterstained with hematoxylin. Serial sections were also subjected to hematoxylin and eosin staining per standard methods to confirm the identity of cells in the region of ISH signals. Images were collected using a Deltavision microscope (Applied Precision), and the fluorescent images were created using softWoRx 5.0 (Applied Precision).

The in situ hybridization assay in this study utilizes branched DNA (bDNA) technology, which offers near single copy mRNA sensitivity in individual cells. The bDNA assay uses a sandwich-based hybridization method which relies on bDNA molecules to amplify the signal from target mRNA molecules. Each probe set hybridizing to a single target contains 20 oligonucleotides pairs. This was followed by sequential hybridization with the final conjugation of a fluorescent dye. Thus, each fully assembled signal amplification ‘tree’ has 400 binding sites for each labeled probe. Finally, when all target specific oligonucleotides in the probe set have bound to the target mRNA transcript, the resulting amplification of signal approaches 8000-fold (20 oligonucleotides times 400 binding sites = 8000 fold).

### Protein antigen detection by immunofluorescence assays

Immunofluorescence staining was performed on fresh frozen cryosections (10 *µ*M thickness) or FFPE sections (5 *µ*M thickness) of mouse skin to visualize the hair follicles present at Day 0 and Day 16. Cryosections were stored at −80°C until use. Cryosections were dried for 30 min at room temperature and fixed by immersion in ice-cold acetone for 10 mins. Cryosections were then air-dried for 5 mins and washed three times with PBS. For FFPE sections, deparaffinzation was performed using xylene and series of alcohol changes. Antigen retrieval for performed using 0.05% trypsin at 37°C for 20 mins. Both cryosections and FFPE sections underwent the same treatment after this step. The sections were blocked for 1 hour using normal donkey serum (NDS, dilution 1∶10; Sigma-Adhrich) in PBS. Sections were then incubated with specific primary antibodies (as described in Table S3) in 1∶5 dilution of blocking solution for overnight at 4°C and then washed three times with PBS. Next, sections were incubated with Alexafluor-488-conjugated donkey anti-rabbit and Alexafluor-594-conjugated donkey anti-goat antibodies (Vector Laboratories, 1∶500) for 1h at 37°C, washed three times with PBS. Final wash was performed with DAPI and the sections were mounted using Flurosav (Calbiochem). Images were collected using a Deltavision microscope (Applied Precision), and the fluorescent images were created using softWoRx 5.0 (Applied Precision). Foxn1 was used as a positive control for matrix cells based on previous literature [50]. Morphology, as determined by brightfield and DAPI staining, was used to identify DP localization. We considered Fgf7 [42] as a positive control for DP localization; however, all antibodies tested showed significant non-specific staining.

### RNA extraction and quantitative Real Time PCR

Total RNA was extracted from mouse skin samples at days 6, 16, 23, 29, 38, 44 and 59 using Agilent's Total RNA isolation mini kit (Agilent Technologies). Reverse transcription reaction was performed with 500 ng of total RNA using the Superscript VILO cDNA synthesis kit (Life technologies). A 1∶25 dilution of cDNA was used in the QRT PCR reaction. QRT-PCR was carried out in a 10 *µ*l reaction mixture with gene-specific primers and *β*-Actin using RT^2^ SYBR Green ROX qPCR Mastermix (Qiagen). The PCR conditions were 95°C for 10 min, and 40 cycles of 95°C for 15 s, 59°C for 30 s, 72°C for 30 s on the ABI HT 7600 PCR instrument. All samples were assayed in quadruplicate. The differences in expression of specific gene product were evaluated using a relative quantification method where the expression of specific gene was normalized to the level of *β*-Actin. Primer sequences available in Supplementary Table S4.

## Supporting Information

Figure S1 Details on the periodic identification by frequency. Probesets were separated into groups based on the frequency of the Principal Periodic Component, a combined group was shown at the far left. (Top) Box plots of the Coefficient of determination, also known as R-Squared values, to indicate goodness of fit. Boxes show the Interquartile range. Results were shown for the complete fit (Fourier Series Regression) and for the simplified fit involving only the Principal Periodic Component. (Bottom) Histogram to show number of probesets at each frequency, not the y-axis is in log scale.
(EPS)

Figure S2Schematic of possible levels of synchronization and the corresponding first and second order parameters. Referring to EQ 9 in the Methods section of the main text, the direction of the arrows corresponds to 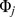 and the magnitude corresponds to 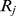. These order parameters would represent the full system and not tracking of individual clusters, noted in the main text by lowercase 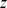 and 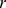. (A) Incoherent state, (B) complete synchronization, (C) asymmetric, out-of-phase, synchronization, (D) symmetric, out-of-phase, synchronization.(EPS)

Figure S3Schematic of a possible two-population expression profile resulting in out-of-phase expression. Here, 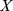 and 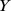 had high and low concentration in pop 1, respectively, and reciprocal concentrations in pop 2. The size of pop 1 was varied while holding the size of pop 2 constant.(EPS)

Figure S4 Negative control for the estimated relative population size in the two-population model. We shuffled the gene expression for each time course so that it did not associate to the hair cycle, see Methods in main text for more details. Importantly, no significant changes in expanding population size were observed with respect to time. The difference between the two time courses may be related to biological or technical batch effect.
(EPS)

Figure S5 Negative control for the two-population model model fits and estimated differential expression. We shuffled the gene expression for each time course so that it did not associate to the hair cycle, see Methods in main text for more details. Considering all probesets, left shows coefficient of determination for 2-population model of expression (negative control for Figure 5A), right shows t-statistic for estimated differential expression (negative control for Figure 5B). Importantly, probesets corresponding to Low Frequency Oscillators (LFO) did not show improved results compared to other probesets.
(EPS)

Figure S6 Two phase histograms shown together with separation determined by different methods. Top (same as in Figure 1C) shows separation directly considering phase. Bottom shows separation determined by the estimated differential expression in the two-population model. Grouping by estimated cell population or by approximated phase produced nearly identical results.
(EPS)

Figure S7 Overlap with hair cell type specific gene signatures. Signatures in the form of probeset IDs were taken from the literature (Lit.) for Matrix (MX), Dermal Papilla (DP), melanocytes (MC) and outer root sheath (ORS) from [36] and Bulge cells (Blg) from [37]. (A) Overlap with this list from both the computationally derived expanding (Exp.) and background (Bkgd.) populations is shown. Corresponding enrichment results are shown in Supplementary Table S1. (B) Overlapping probesets between the two experimentally derived signatures.
(EPS)

Figure S8 Quantification of hair growth and validation of selected microarray results by QRT-PCR. Two biological replicates are shown (points), for visual assistance a line is drawn through the mean of each replicate. (A) Melanogenesis graph for samples at time points for which QRT-PCR was performed. High scores were indicative of anagen. Both biological replicates showed the same behavior. (B) QRT-PCR analysis for background, DP enriched, candidate genes. A cyclic pattern in the expression was observed with low expression in the mid to late anagen phase and increasing in telogen onset (day 29), a slight decrease was observed in late telogen. (C) QRT-PCR analysis for matrix derived cell candidate genes. Maximum expression was observed during the anagen phase (day 23) and the expression declined to a minimum from catagen to telogen phase (Day 29 to 39). The reaction for each biological replicate was performed in quadruplicate (average was reported) and normalized to *β*-Actin.
(EPS)

Figure S9 Technical controls for RNA imaging by In Situ Hybridization (ISH). We show two replicates of both negative, in the absence of any RNA probe, and positive, addition of the Ubiquitin C (UBC) RNA probe, controls for both day 0 and day 16 time points. UBC was the positive control suggested by the manufacturer. STAT5A was added to positive controls for comparison purposes.
(TIF)

Figure S10 Expression trajectories that matched the criterion for possible drivers of negative feedback. Probesets identified as low frequency oscillators and increased expression near catagen onset that was not captured by a population model with static intracellular expression. The 88 expression signals meeting this criterion are shown relative to the static population model, for example values above one indicate increases above what could be expected by static intracellular expression.
(EPS)

Figure S11 Similar to Supplementary Figure S1, we show the error between the estimated Fourier Series to the actual data. Here the error measurement used was median absolute deviation (MAD), which may be more suitable considering the model was estimated using robust regression. We show standard Interquartile range box plots for MAD over all oscillators as well as individual frequencies.
(EPS)

Figure S12 Similar to Figure 2A in the main text, we show the magnitude of the first and second complex order parameter for the hair cycle system. Figure 2A was limited to only low frequency oscillators; however, here we show results that included all gene expression profiles identified as periodic. Even considering all oscillators, we still observed asymmetric, out-of-phase synchronization. This pattern dominates the expression due to the size of the low frequency oscillating group.
(EPS)

Figure S13Comparison simulations of the high and low dimensional coupled oscillator systems. The high dimensional system simulates all 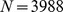 individual oscillators (see EQ 10 in Methods of the main text) and the low dimensional system simulates only the order parameters corresponding to the two clusters (see EQ 12 in Methods of the main text), which is technically valid as 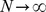. Here, we considered the stable coupling configuration from Figure 3A, red solid line. Given the uncertainty of the high dimensional system (unknown initial conditions of individual oscillator before synchronization) we show the 1 standard deviation envelope about the mean in the shaded blue region, as determined over 100 simulations with random initial conditions. The high dimensional system of individual oscillators has the same behavior as the low dimensional system, and most importantly, we observed little quantitative variation at long times.(EPS)

Table S1 Cell type enrichment for model populations. P-values derived from hypergeometric distribution to test enrichment of cell type specific probesets from lists reported in the literature [36], [37]. Fraction indicates number of overlapping probesets relative to the total reported cell type specific probesets.
(PNG)

Table S2 Target probe set information.
(PNG)

Table S3 Immunofluorescence antibody information.
(PNG)

Table S4 QRTPCR primer information.
(PNG)

File S1 Periodic genes with a Normalized Google Distance (NGD) to the term ‘hair’ of less than one for abstracts in PubMed. Genes were considered periodic if they had a symbol that mapped to at least one probeset shown in figure 1A. As mentioned in methods NGD<1 is indicative of a better than random chance of term co-occurrence.
(TXT)

File S2Movie showing the trajectories of individual oscillators in the coupled oscillator system (for details see EQ 10 in Methods of the main text and section *Expression modeled as a system of coupled oscillators*). Oscillators were started from a random, incoherent state and parameter values for the stable configuration were used (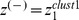; related to figures 3A and S12). As in Figure 1C, green and blue was used to indicate cluster 1 and 2, respectively.(ZIP)

File S3 Table of probesets considered as low frequency oscillators (see section *Identification and characterization of periodic expression signals*) and estimated to have differential expression between the expanding and background populations (see section *Associating gene clusters to hair specific cell populations*). Statistics and metrics, described in the methods, were included.
(XLSX)

File S4 List of gene symbols corresponding to possible negative feedback targets (see section *Identification of negative feedback targets*).
(TXT)

File S5 GO term enrichment for gene symbols corresponding to possible negative feedback targets (see section *Identification of negative feedback targets*).
(XLSX)

File S6 Compressed folder of code and scripts used in this study (see contained readme.txt).
(ZIP)
