# Supplementary figures and images for: Mouse Hair Cycle Expression Dynamics Modeled as Coupled Mesenchymal and Epithelial Oscillators
Source: PLoS Comput Biol. 2014 Nov 6;10(11):e1003914. doi: 10.1371/journal.pcbi.1003914 (PMC4222602; doi:10.1371/journal.pcbi.1003914)

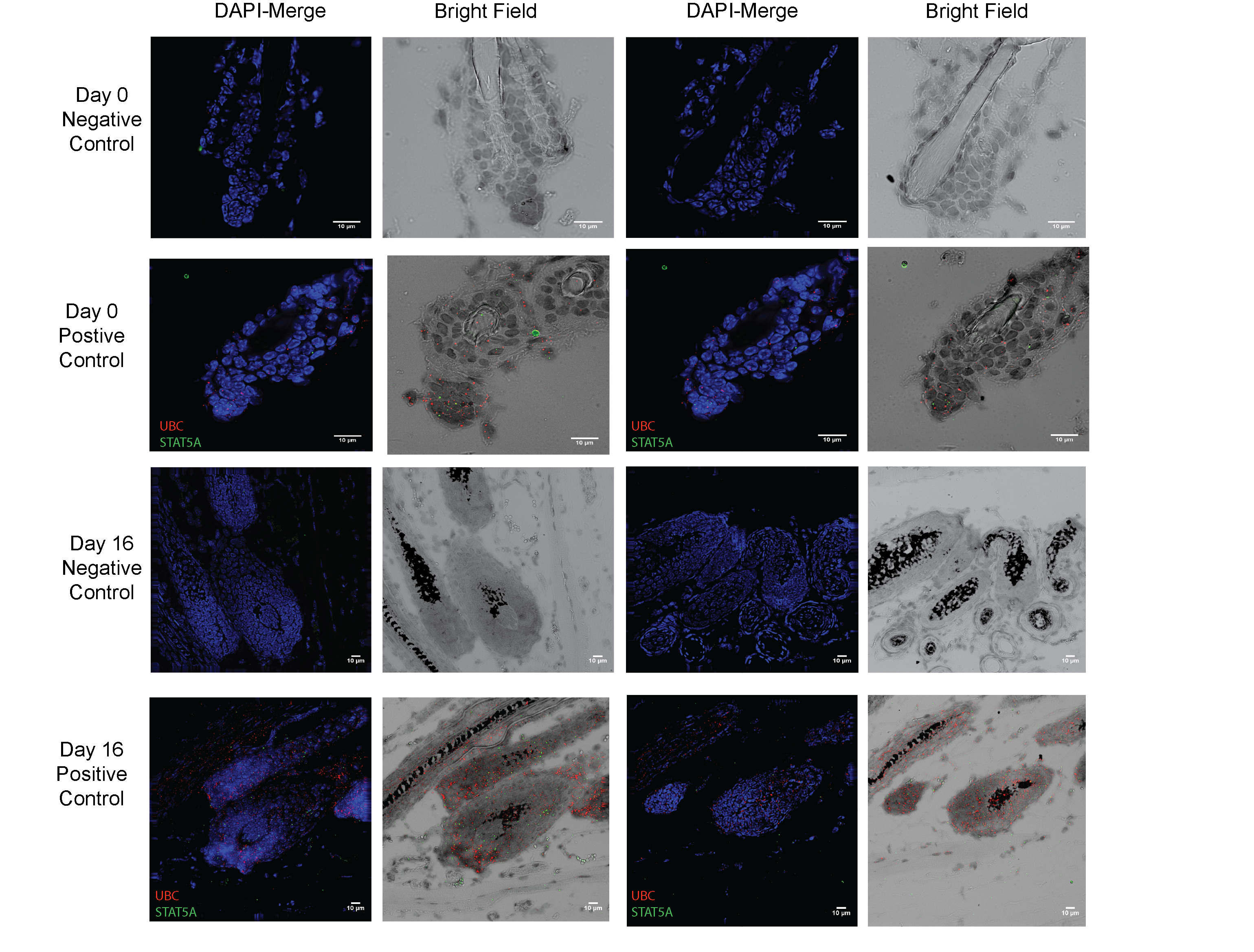

Supplement: Figure S9 — Technical controls for RNA imaging by In Situ Hybridization (ISH). We show two replicates of both negative, in the absence of any RNA probe, and positive, addition of the Ubiquitin C (UBC) RNA probe, controls for both day 0 and day 16 time points. UBC was the positive control suggested by the manufacturer. STAT5A was added to positive controls for comparison purposes. (TIF) [file pcbi.1003914.s009.tif]

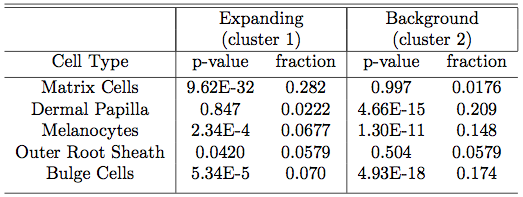

Supplement: Table S1 — Cell type enrichment for model populations. P-values derived from hypergeometric distribution to test enrichment of cell type specific probesets from lists reported in the literature [36], [37]. Fraction indicates number of overlapping probesets relative to the total reported cell type specific probesets. (PNG) [file pcbi.1003914.s014.png]

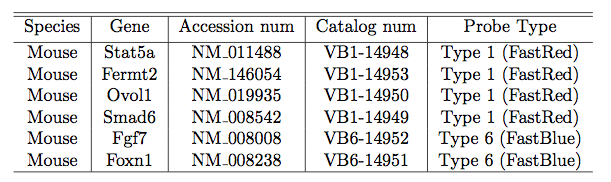

Supplement: Table S2 — Target probe set information. (PNG) [file pcbi.1003914.s015.png]

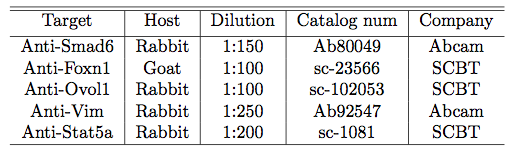

Supplement: Table S3 — Immunofluorescence antibody information. (PNG) [file pcbi.1003914.s016.png]

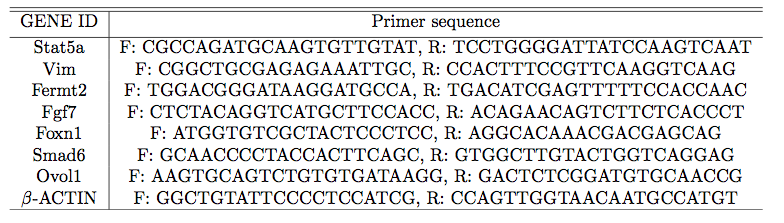

Supplement: Table S4 — QRTPCR primer information. (PNG) [file pcbi.1003914.s017.png]
